# Supplementary figures and images for: An integrated model for predicting KRAS dependency
Source: PLoS Comput Biol. 2023 May 4;19(5):e1011095. doi: 10.1371/journal.pcbi.1011095 (PMC10187917; doi:10.1371/journal.pcbi.1011095)

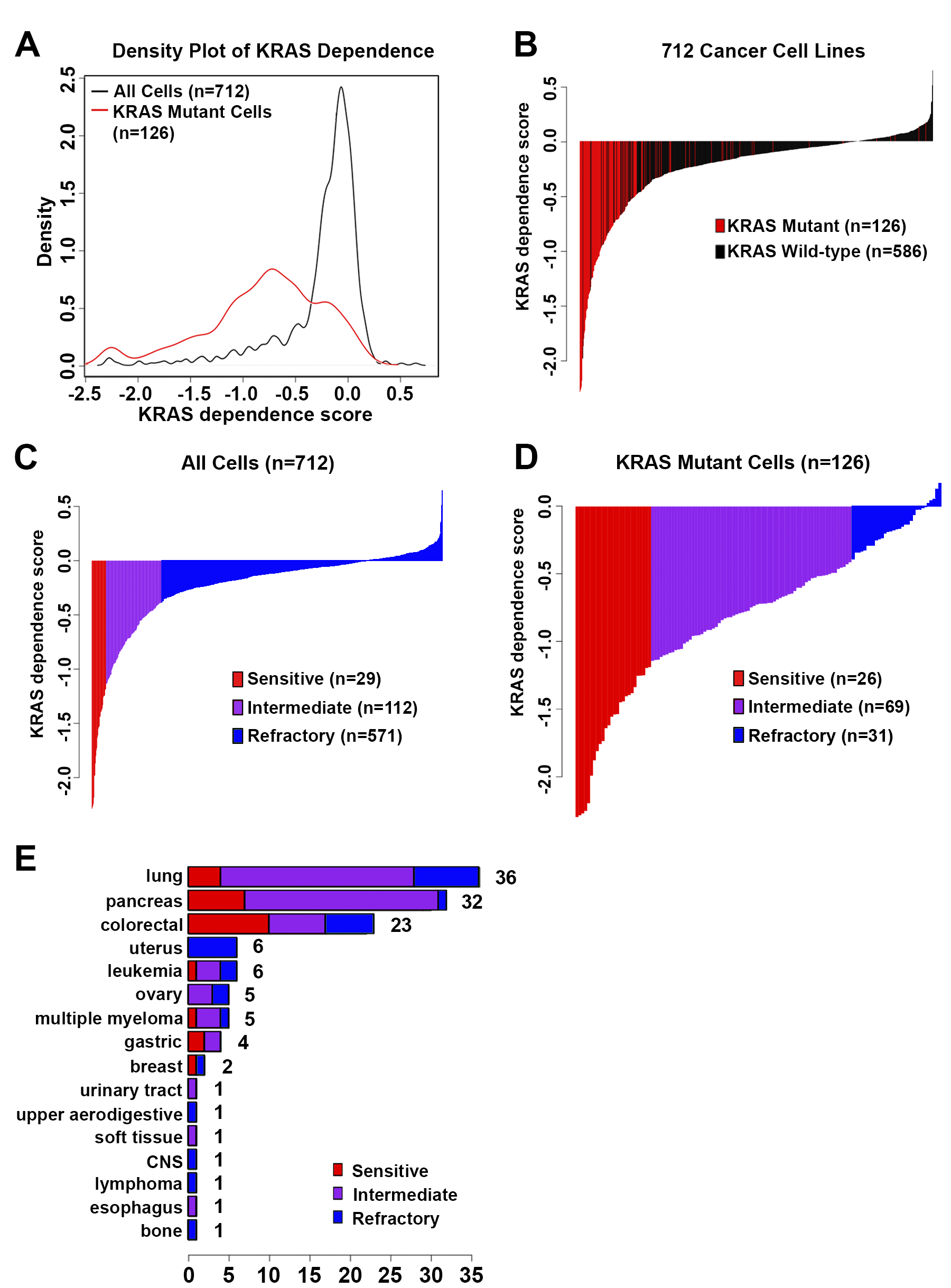

Supplement: S1 Fig — (A) Density plot of KRAS dependency score in all 712 cancer cell lines (black line) and in 126 KRAS-mutant lines (red line), p-value = 6.71e-30 (Wilcoxon rank sum test). (B) Waterfall plot of KRAS dependency scores, color-coded by KRAS mutation status. Red and black color bars represent KRAS-mutant and wild-type cells, respectively. (C) Waterfall plot of KRAS dependency scores, color-coded by KRAS mutation k-mean cluster (k = 3). Red, purple and blue color bars represent sensitive, intermediate, and refractory clusters, respectively. (D) Waterfall plot of KRAS dependency scores in KRAS-mutant cells only. (E) Bar plot of disease types in 126 KRAS-mutant cell lines. (TIF) [file pcbi.1011095.s001.tif]

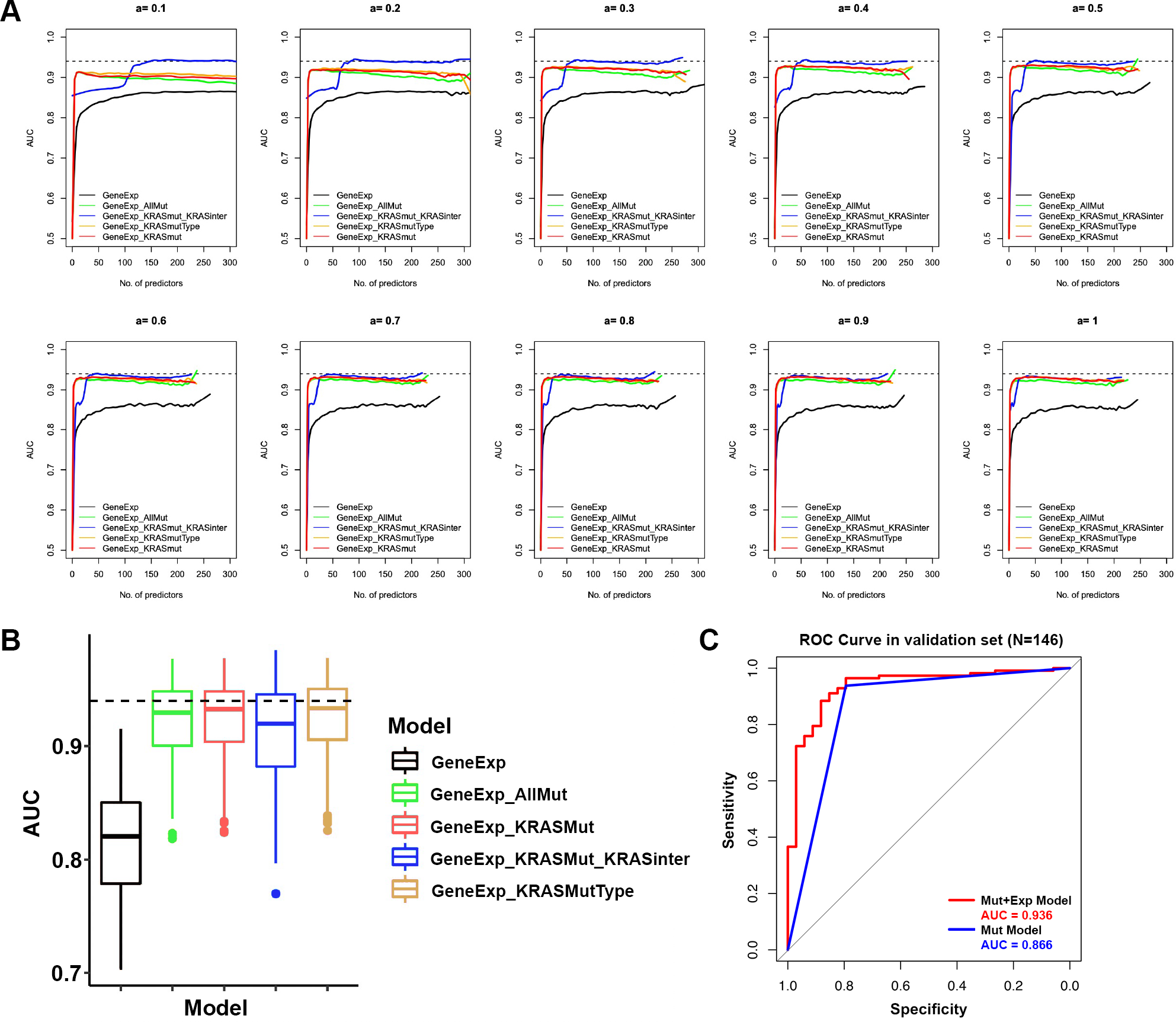

Supplement: S2 Fig — (A) Performance comparisons in the training set among the five different feature sets and in different alpha (0.1 to 1) were shown in each plot for AUC versus the number of features. Different colors of lines represent the five different feature sets: gene expression only in black, gene expression and mutation in green, gene expression and KRAS mutation status in red, gene expression and KRAS mutation type in orange and gene expression, KRAS mutation and interaction term between them in blue. The black dotted line is at the highest AUC = 0.94 for easy comparison among plots. (B) Boxplot of AUC comparison at alpha = 0.9 and number of features between 15 to 25 during the MCCV. The black dotted line is at the highest AUC = 0.94. (C) Receiver operating characteristic (ROC) curve comparing the model performance between the K20 model (in red) and the KRAS mutation only model (in blue) in the validation set. (TIF) [file pcbi.1011095.s002.tif]

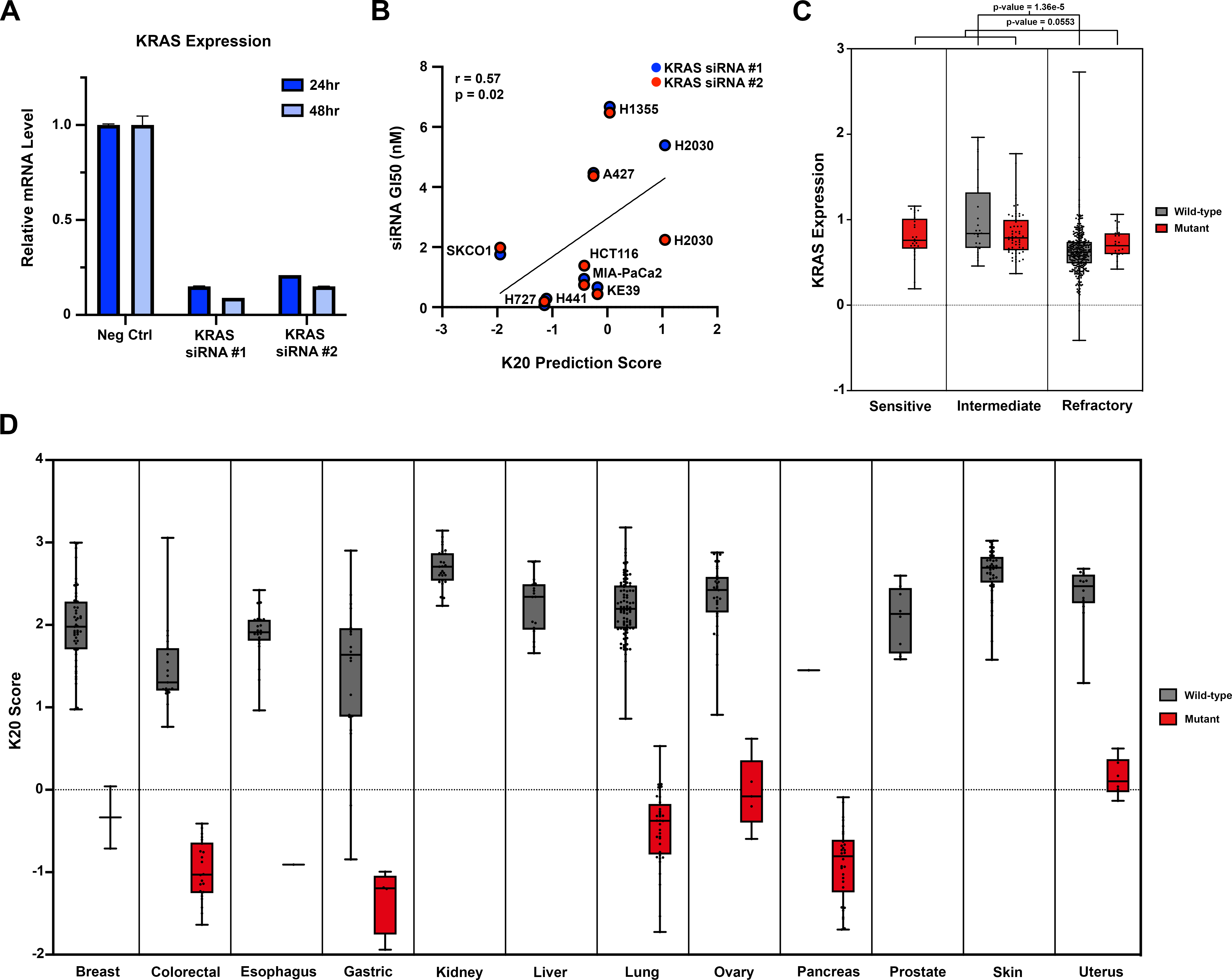

Supplement: S3 Fig — (A) Relative KRAS expression by RT-qPCR in HCT116 cells following treatment with the control siRNA, KRAS siRNA #1, and KRAS siRNA #2 at 20nM for 24 and 48 hrs. Error bars indicate SEM. (B) Scatter plot of K20 prediction scores (in x-axis) vs. GI50 scores of the two KRAS siRNAs treated in nine different cancer cell lines (in y-axis) shows correlation (Spearman r = 0.57, p = 0.02). (C) Box plot of KRAS expression by KRAS dependency class and mutation status. Wilcoxon p-values were shown for two group comparisons. (D) Box plot of K20 prediction scores in 12 CCLE cancer types overlapped with the 14 TCGA solid tumors, separated by disease type and KRAS mutation status. Each sample is represented as a dot. (TIF) [file pcbi.1011095.s003.tif]

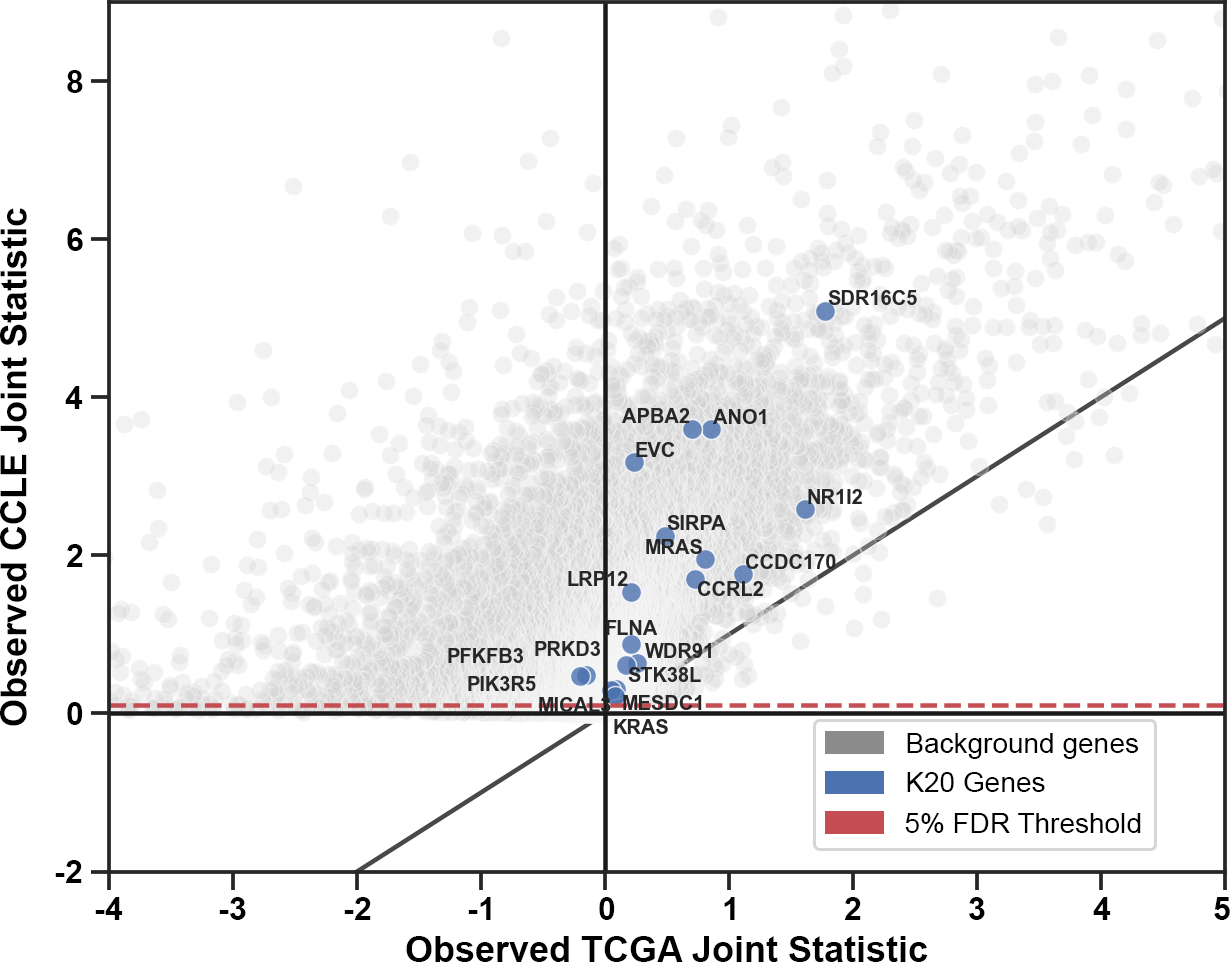

Supplement: S4 Fig — The observed TCGA and CCLE joint statistics of all genes are shown along the x- and y-axes, respectively. The 19 genes included in the K20 model are shown in blue, while the rest of the genes are shown in grey. The FDR threshold was set to 5% for CCLE joint statistic, which was equal to 0.106 here (shown as a red dotted line). (TIF) [file pcbi.1011095.s004.tif]

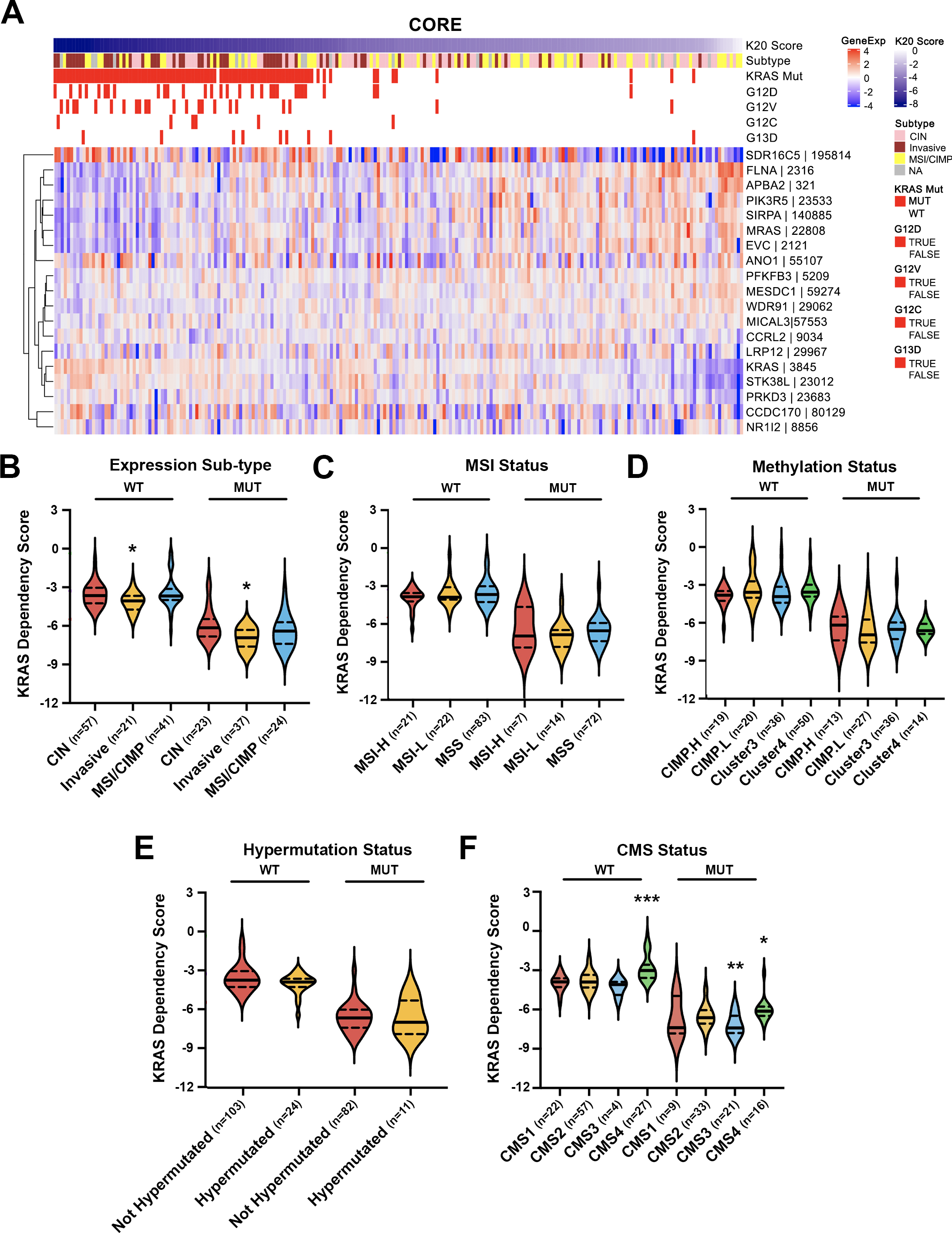

Supplement: S5 Fig — K20 model predicts that most of the colorectal cancer patients with KRAS mutation are KRAS-sensitive with some exceptions and the model prediction scores are associated with several TCGA expression subtypes. (A) Heatmap showing gene expression of 19 classifier genes and KRAS mutation status and types in TCGA colorectal cancer patients. Samples were sorted by prediction scores (left to right: lowest to highest). (B-E) Violin plots of K20 model prediction scores by (B) expression subtypes (KRAS-wildtype (WT): invasive vs CIN, p = 0.0228; invasive vs MSI/CIMP, p = 0.0097; KRAS-mutant (MUT): invasive vs CIN, p = 0.0022), (C) MSI status, (D) methylation status, (E) hypermutation status, and (F) CMS status (KRAS-wildtype (WT): CMS1 vs CMS4, p<0.0001; CMS2 vs CMS4, p<0.0001; CMS3 vs CMS4, p = 0.0004; KRAS-mutant (MUT): CMS2 vs CMS4, p = 0.0262; CMS3 vs CMS4, p = 0.0003; CMS2 vs CMS3, p = 0.0062). Wilcoxon rank-sum test was used for comparisons. (***) = p<0.001, (**) = p<0.01, (*) = p<0.05 (TIF) [file pcbi.1011095.s005.tif]

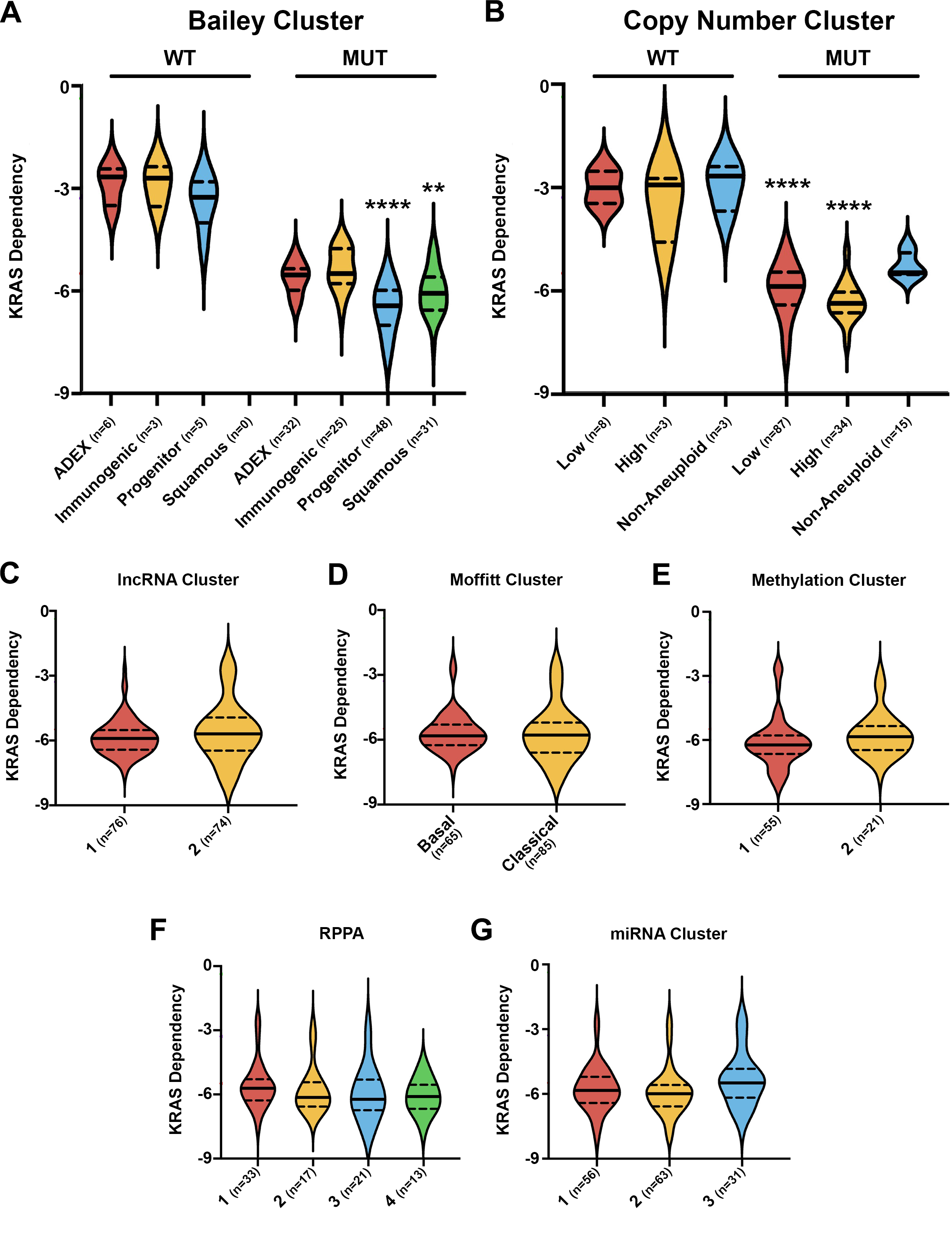

Supplement: S6 Fig — Violin plots of K20 prediction score split into KRAS-wildtype (WT) and KRAS-mutant (MUT) groups by (A) Bailey cluster (MUT: progenitor vs ADEX, p<0.0001; squamous vs ADEX, p = 0.0046, progenitor vs immunogenic, p<0.0001; squamous vs immunogenic, p = 0.0003; squamous vs progenitor, p = 0.0107) and (B) copy number cluster (MUT: low vs non-aneuploid, p<0.0001; high vs non-aneuploid, p<0.0001; low vs high, p = 0.0051). Additional violin plots of K20 prediction scores by (C) lncRNA cluster, (D) Moffitt cluster, (E) methylation cluster, (F) RPPA, and (G) miRNA cluster. Wilcoxon rank-sum test was used for comparisons. (****) = p<0.0001, (**) = p<0.01 (TIF) [file pcbi.1011095.s006.tif]

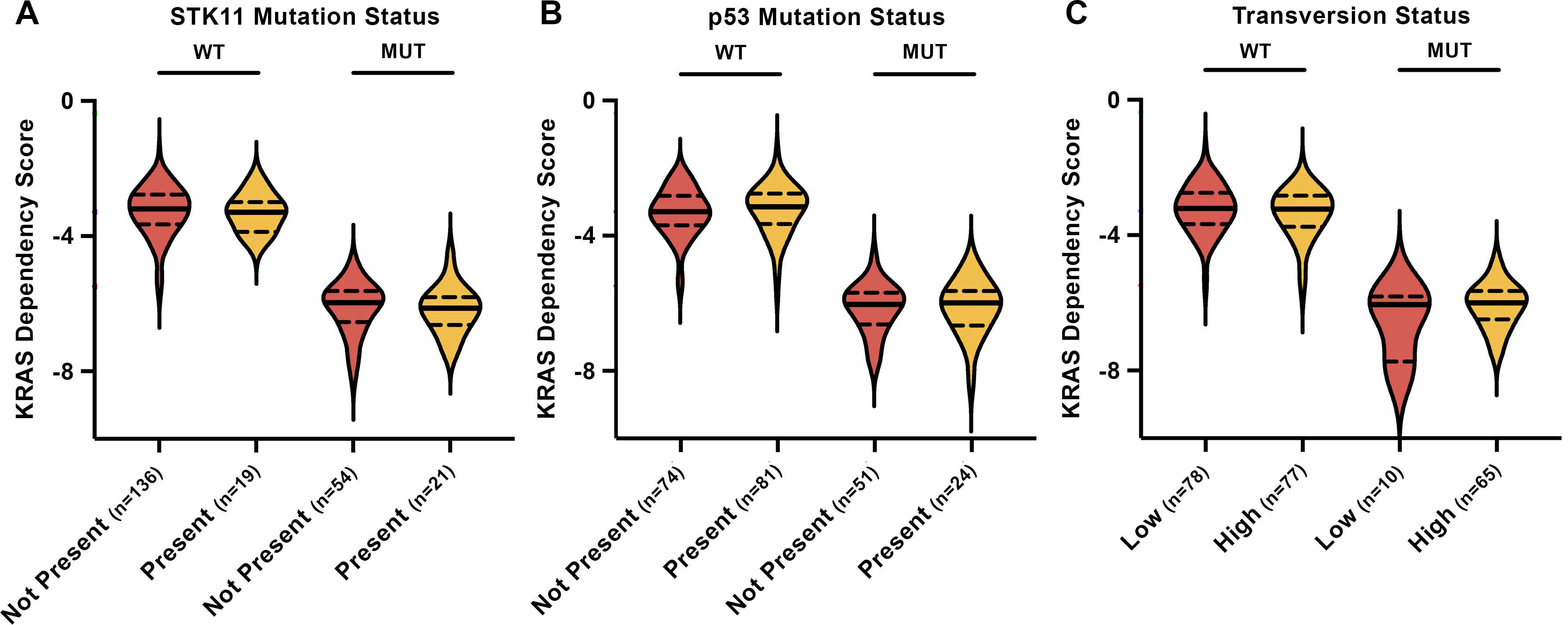

Supplement: S7 Fig — Additional violin plots of K20 prediction scores split into KRAS-wildtype (WT) and KRAS-mutant (MUT) groups by (A) STK11 mutation status, (B) p53 mutation status, and (C) transversion status. Wilcoxon rank-sum test was used for comparisons. (TIF) [file pcbi.1011095.s007.tif]
