# Supplementary material for: An integrated model for predicting KRAS dependency
Source: PLoS Comput Biol. 2023 May 4;19(5):e1011095. doi: 10.1371/journal.pcbi.1011095 (PMC10187917; doi:10.1371/journal.pcbi.1011095)
Supplement: Attachment — Submitted filename: Response to Reviewers_221216.docx [file pcbi.1011095.s009.docx]

Dear Colleagues:

We thank the reviewers for their positive and constructive comments. We have addressed the concerns raised by the reviewers where experimentally possible. Changes from the original version are highlighted in the revised version of the manuscript and in the figures. Point-by-point summary follows:

**Response to reviewer comments**

**Reviewer 1:** The manuscript by Tsai et al presents a proposal for a prognostic panel of 20 markers (K20) for sensitivity to KRas modulation across multiple cancer types. KRas mutation is surely significant across human cancer and although recently druggable, still present major clinical challenges. There is precedent for similar types of marker panels such as the PAM50 set for breast cancer. The authors integrate large publicly available datasets in potentially new and what seems to be interesting ways to arrive at their K20 panel that is claimed to be predictive of KRas dependency.

We appreciate the positive comments of the reviewer as well as their constructive suggestions.

Major decisions are also made in a rather ad hoc manner, such as the stratification into three KRas dependency groups although the data form a continuum---this is not necessarily a problem but the reader is left wanting for more discussion and justification. Also other feature sets with better AUC are left behind.

We thank the reviewer for bringing up this important point. We followed the methods previously described in McDonald et al (Cell, 2017) to apply k-mean clustering (k=3) and determine the stratification of the dependency groups. We have highlighted this in the methods section (page 24, lines 663-666). Additionally, we chose this feature set because of its simplicity and performance in comparison to other sets. To emphasize this point, we have added text in page 9, lines 308-310 as well as an additional supplementary panel (Panel C in Fig S2).

The authors apply the K20 panel to TCGA data. While application to clinical data from TCGA on its surface seems useful, this Reviewer is unsure how much scientifically is truly added with these applications, since there seems to be no KRas dependency data in TCGA that could be used to test the K20 model predictions.

(1) The statement is made that attempts to block downstream MAPK signaling has not yet proven to be clinically effective. However what about MEK and Raf inhibitors? This section needs more nuance and balance.

We appreciate this important point and agree that including nuanced information on downstream MAPK inhibitors can provide important context. We have added information on MEK and Raf inhibitors (and their limitations, which includes the development of resistance) to the introduction (page 3, line 74-77).

(2) The methods section in general would benefit from far more detail. For example, details of how CCLE data was “aligned” with TCGA data seem critical but it is not clear nor reproducible from what is written. In the section on K20 model building, what is interaction with KRAS mutation status? For the CCLE and TCGA joint statistic calculation, why is there a max variance (how are there several?)? Treatment of cells for siRNA experiments is also lacking detail, such as when siRNA was added, how long cells were given to attach, what kind of plates were used, and so on.

We have revised the methods section to include more detail and improve clarity and readability.

We have added formulas (page 25, line 685-689) to explain how the adjusted prediction score for TCGA was calculated. For the gene expression and KRAS mutation interaction model, each gene has two features to be included in the ElasticNet model. One is the original expression value, and one is the mutation status (1 for KRAS mutation, 0 for KRAS-WT) multiplied to the expression value. This is so that way we can model different effects of KRAS expression depending on its mutation status.

We apologize for any confusion regarding the joint statistic calculation. We have updated the method section to avoid confusion.

(3) Results page 12, what is alpha and why is it significant that it takes different values with different feature sets? How was binary classification accomplished with the expression levels?

Alpha and lambda are the two tuning parameters in ElasticNet (regularized linear regression). We have added more detail in the methods section: page 25, line 694-697.

After applying the K20 model, each sample has one prediction score, and based on the training data, we can define a threshold to determine their binary classification (i.e. smaller or negative scores indicate higher sensitivity, so if the threshold is zero, samples with negative prediction scores are predicted to be non-refractory).

(4) The authors discuss that KRas expression levels are by far the highest contributor to the K20 panel outside of mutation status (Fig. 1C). Are the other 18 expression levels even needed?

We thank the reviewer for this comment. Because KRAS expression is highly correlated with KRAS mutation status, a model using only KRAS expression and KRAS mutation would not outperform our K20 model. If we only pick two features, the top two would be KRAS mutation status and MRAS expression.

We have compared the K20 model with multiple feature combinations. Their performance AUC’s in the validation set are listed below:

K20 model (20 features): 0.94

KRAS mutation status only (1 feature): 0.87

KRAS expression only (1 feature): 0.72

KRAS mutation status and KRAS expression (2 features): 0.75

KRAS mutation status and MRAS expression (2 features): 0.90

(5) Fig. 2D is the main experimental validation summary but it is unclear how these siRNA dose response experiments support the claims that the K20 panel has predictive power for KRas dependency. Maybe a plot of sensitivity vs. K20 score like 2E could demonstrate this better.

We thank the reviewer for this suggestion. We have included a correlation plot between predicted K20 score and experimental GI50 of both KRAS siRNAs in the six tested cancer cell lines in Supplementary Figure 3, with a correlation coefficient of 0.62.

**Reviewer 2:** Tsai and colleagues seek to predict response to KRAS G12C inhibitors by generating a score (K20) from some 400 cell lines in DEMETER2 and validating it using TCGA and other data. The K20 model embeds several genes that are known in the field and by orthogonal approaches to be linked to KRAS signaling, which appears highly reasonable.

We appreciate the positive comments of the reviewer as well as their constructive suggestions.

(1) A suggested improvement would be to generate the K20s for one tumor type at a time for colorectal, lung and pancreas cancers in the discovery part of the project, as that would potentially reduce background from analyzing different tumor types in the same score and result in a better performing score for each tumor type.

We thank the reviewer for this suggestion. Due to the small sample size, we have tried modeling specifically with cancer type information during the training process, but it did not improve the model performance. When more data within each cancer types becomes available, generating a model for each cancer type may become feasible and a cancer type-specific model may have better performance than one generalized model.

(2) The text relating to figs 3-5 feels repetitive/cataloguing and should be condensed into a single display item/figure panel with more concise text.

We appreciate the reviewer comment and acknowledge that the text in figs 3-5 may seem repetitive. This model of KRAS dependency introduces genes that may be potential biomarkers for patients who may benefit from KRAS inhibitors. Because each cancer sub-type has different sub-classifications without significant overlapping characteristics, we respectfully suggest that they cannot be combined without minimizing important insight. A practical example of this that is already being observed clinically is the distinct differences in response rates to KRAS G12C inhibitors between lung adenocarcinoma and those with colon carcinomas.

We hope that the differences we have highlighted can help further distinguish between patients who may respond to KRAS inhibition, and those who are unlikely to do so.

**Reviewer 3:** In their manuscript entitled “An Integrated Model for Predicting KRAS Dependency”, the authors developed a model to predict KRAS dependency, and externally validated the performance of the model using genetic depletion assays and an external dataset. In addition, they applied the model to TCGA pan-cancer datasets. This study provides a model by integrating expression, mutation data and RNAi screening data to predict KRAS dependency, for improving the stratification of KRAS mutant tumors to benefit the therapies of KRAS inhibitions. This work is valuable from the perspective of precision medicine of KRAS mutant tumors.

We appreciate the positive comments of the reviewer as well as their constructive suggestions.

(1) To help the readers better understand this study, the authors are suggested to explain the term “kras dependency score” where it first appears in the manuscript.

We thank the reviewer for this comment. To improve readability, we have defined KRAS dependency score in the results section (page 6, lines 131-132).

(2) There are several similar terms in the manuscript, such as k20 predictive score, K20 prediction score, and predicted k20 score. If these terms have the same concept, they are suggested to have a consistent naming. Also, explain this term in the first place where it appears.

These terms refer to the same concept. We have revised the manuscript to standardize this term to K20 prediction score and defined it in page 10, lines 349-350.

(3) When the authors used a cutoff to define the cell lines to be “sensitive” or “refractory”, how to use a reasonable cutoff? In external validation of the model, they used 1.477 as a cutoff. However, no information about the cutoff was given in the training model. Is the cutoff 1.477 consistent with the training model? Moreover, is this value reasonable to distinguish “sensitive” from “refractory” from the perspective of biological meaning?

We apologize for the confusion. In the cell line data, we took the same method as described in E. McDonald et. Al (k-mean clustering), where k is 3, to define the 3 clusters (i.e. sensitive, intermediate and refractory) and further define a binary classification, which combines sensitive and intermediate clusters into a non-refractory group. In the binary classification model, we identified threshold of 1.477 has the highest sensitivity and specificity in the DRIVE dataset (our training set), so it was selected as the threshold for external validation.

(4) The authors used five different features to develop the model. I don’t well understand the feature set “the interaction between KRAS mutation status”. This feature set can be described with more details.

We apologize for the confusion. For the gene expression and KRAS mutation interaction model, each gene has two features to be included in the ElasticNet model. One is the original expression value, and one is the mutation status (1 for KRAS mutation, 0 for KRAS-WT) multiplied to the expression value. This way, we can model different effects of KRAS expression depending on its mutation status.

(5) In page 12 line 264, gene expression index was calculated by reads per kilobase normalized on log2 scale. I’ m wondering if gene expression index was measured by FPKM, TPM or others in the manuscript? Was gene expression also normalized by sequencing depth?

We apologize for the confusion. Gene expression is normalized by sequencing depth, but not gene size. It is RPKM and log2 transformed.

(6) The Fig. 2A and 2B actually don’t provide the information of “sensitivity of 0.95 and specificity of 0.89” which is described in the manuscript. In Fig. 2C, though the model prediction scores are highly correlated with the KRAS dependency scores, the prediction scores in y-axis actually can’t distinguish “sensitive”, “intermediate” and “refractory” very well.

We apologize for the confusion. The ROC curve was shown in Supplementary Figure 2C. We have corrected the text now to better reflect this. We now realize the large sizes of the dots in Figure 2C could be misleading. If we compare the prediction score of non-refractory to refractory, it is significantly lower (p<2.2e-16, violin plot is shown below). To help better reflect these distinctions, we have updated Figure 2C with smaller dot sizes.

(7) In the model, KRAS mutation and KRAS expression have the most contribution on KRAS dependency score. Beyond these well-known biomarkers, more efforts should be made to investigate the other features in the model to understand the KRAS independent tumors for developing the new strategies of precision medicine.

We appreciate the reviewer comment and acknowledge that there is limited information on how the other features in our K20 model may relate to KRAS independence. In the discussion section, we have speculated on how these genes may be related to KRAS. For example, STK38L expression is associated with KRAS dependency in our model and is frequently co-amplified with oncogenic KRAS. In contrast, FLNA is often down-regulated in KRAS-mutant tumors and is associated with KRAS tolerance in our model, indicating that higher expression of FLNA in tumors may correlate to reduced efficacy of KRAS inhibitors. We have acknowledged these patterns in the discussion section and emphasized the importance of future experimental validation of our findings to find potential targetable vulnerabilities for synergistic treatments with KRAS inhibitors.

(8) In Fig. 1A, the information of “kras mutant” and “sensitive” is shown in the same red color. The different colors can be used in the same figure panel. In addition, red and green colors in the same panel can be replaced with other colors. In Fig. 1C, add the name of x-axis. In Fig. 1D, what does the number after the gene name represent for? This could be added in the figure legend.

We thank the reviewer for this comment. We have updated our figures (including Figure 1A) to remove red and green in the same plot. We also removed the red color from the KRAS dependence score bar plot and used additional color bars to represent mutation status for KRAS and other RAS and Raf family genes. The number after each gene name in Figure 1D represents its Entrez ID. This detail has been added to the figure legend for Figure 1.

**Reviewer 4:** Tsai, et al, describe a model based on gene expression and mutational status to predict KRAS dependency of tumors. The model is trained on cell lines and validated on TCGA and in vitro dose response experiments.

(1) The concept of modeling RAS dependence using gene expression data is not a new one: see (Singh, Cancer Cell, 2009; Way, Cell Reports, 2018; Guinney, Clinical Cancer Research, 2013). Authors need to clearly articulate the advantage/comparison of their approach over prior models.

We thank the reviewer for this comment. We believe our model is the first to integrate multiple datasets that can relate KRAS dependencies, genomic features, and tumor-specific transcriptional profiles in order to generate a signature for KRAS dependency across all cancer types. While Singh et al (2009) and Guinney et al (2013) focused on lung, pancreatic, and colorectal cancers, our model integrated data from other carcinomas such as breast, skin, etc. In contrast to Way et al (2018), our model was built on DEMETER2 dependency data, which adds an additional layer of complexity beyond RAS pathway activation and/or events which may phenocopy it. We have clarified this advantage in the introduction in page 4, lines 112-115.

(2) KRAS activating mutations are not defined. Is any amino acid concerned considered, or only those with canonical codon changes?

We thank the reviewer for this comment. We have now included the KRAS activating mutations in the figure legend of figure 1A, also shown below:

KRAS: A146A, A146T, R164, T74P (+ any mutations at G12, G13, Q61)

BRAF: L597R, V226M, G466V, R682Q, G464E, N581Y, G464V, G469A, L597V, G596R (+ any mutations at V600)

NRAS: A146V, E132K (+ any mutations at G12, G13, Q61)

HRAS: T58T ((+ any mutations at G12, G13, Q61)

In the cell line training dataset, all 106 except four of the KRAS mutations were included in the activating mutation list. The KRAS mutation types include the following:

p.A146T

p.G12A

p.G12C

p.G12D

p.G12R

p.G12S

p.G12V

p.G12C

p.G13C

p.G13D

p.P121H (not activating, exist in one cell line)

p.P140H (not activating, exist in one cell line)

p.Q61H

p.V14I (not activating, exist in one cell line)

p.Q61K

p.Q61L

p.R164*

p.R97I (not activating, exist in one cell line)

p.T74P

p.V160A

(3) How do the authors justify the exclusion of NRAS / HRAS from their model?

We thank the reviewer for this thoughtful question. As KRAS G12C inhibitors have now been FDA approved, and potential G12D-specific and pan-KRAS inhibitors are also entering the clinic, we chose to build a model on KRAS dependency specifically to determine potential biomarkers that can be used to select patients across all tumor types for precision medicine. Although NRAS and HRAS mutations may also support our model, these mutations are more limited in their spectrum of cancer types; namely, NRAS mutations mainly occur in melanoma (a non-carcinoma with unique transcriptional states), and HRAS mutations mainly occur in head and neck cancers. For these reasons we have not included NRAS and HRAS mutations in our model.

(4) Fig 1A needs to overlay other activating RAS mutations or those that may phenocopy activation of this pathway (NRAS, HRAS, BRAF). This applies to Fig 3A as well, which by focusing exclusively on KRAS may be missing activated samples from NRAS and/or BRAF, esp in GI cancers but others as well. In Line 340/341, the authors attribute this to false negative KRAS mutations, but is more likely to be explained by alternative RAS activating mutations.

We thank the reviewer for this insightful comment. We acknowledge the potential of mutations which may phenocopy KRAS and have now included those activating mutations in Figure 1A and Figure 3B, which largely demonstrates these are mutually exclusive with KRAS and not well correlated with KRAS dependency.

(5) “we rescaled the K20 prediction scores of TCGA-PANCAN samples to align with CCLE cell lines so that the adjusted TCGA-PANCAN prediction scores have the same minimal and maximal values as CCLE prediction scores.” This is an important point that seems to be glossed over, I would like some more detail on the approach and justification for this. Aligning the max and min could be sensitive to outliers, and I’m also worried about tissue specific effects and the two cohorts not being matched in proportion of samples from each tissue type.

We thank the reviewer for this comment. We added formulas (page 25 line 686) to explain how the adjusted prediction score for TCGA was calculated. To avoid the selection of different tissues, we have excluded TCGA cancer types that were not represented in the CCLE training set.

(6) ‘gene expression of genes with standard deviation (SD) large than 0.5’ large -> larger

This typo has been corrected.

(7) In Fig 1B the AUC of the continuous output of the Mut+Exp Model is compared to the AUC of a binary Mut Model. This is misleading because the AUC of a binary model will very often be lower because the ROC curve only has 3 points with the curve interpolated between them. It would be most convincing to compare binary classification scores between the models using the threshold of the Mut+Exp Model selected for external validation.

We apologize for the confusion. Supplementary Figure 2B shows the ROC curves for the validation set only. If we select a threshold using the training set only, the K20 model would have a sensitivity of 0.853 and specificity of 0.884 in its validation set, while the KRAS mutation model would have a sensitivity of 0.794 and specificity of 0.938.

(8) “we only included genes that had variations (SD>0.5 in log2 normalized gene expression) in the lung adenocarcinoma (LUAD), pancreatic adenocarcinoma (PDAC), and colorectal cancer (CORE)” How many genes was this? I’d be curious to know the number of possible features of all the compared models.

Using genes with expression variation in tissues reduced gene number from ~2K to 9652 genes.

Here are the number of features included in each model:

Gene expression only: 9652

Gene expression + gene level mutation status: 26344

Gene expression + KRAS mut status: 9653

Gene expression + KRAS mut type: 9673

Gene expression + KRAS mut + KRAS interaction: 19305

(9) “We selected the feature set with “gene expression and KRAS mutation status” as our final model because of the minimal performance difference (i.e., AUC=0.94 vs. 0.93) and the smaller number of features required” I agree with the parsimonious approach of selecting a simpler model with similar performance. The justification here and in Fig S2A, however, is a bit weak. This is partially due to Fig S2A being difficult to read. Showing the standard error of the AUC from the cross validation could strengthen this by showing that the simpler model does not have significantly worse performance.

We apologize for the unclear visualization. We added an AUC boxplot comparison among feature sets in Supplementary Figure 2C specifically for the selected alpha value and restricted to a small number of features (15 to 25). As the difference among feature sets 2, 3 and 4 was very minimal, we chose the simplest model, which was “Gene expression + KRAS mutation”.

(10) “Unexpectedly, aside from KRAS mutational status, we found that KRAS expression itself played the strongest role in predicting KRAS dependency (Fig. 1C)” - I don’t think this is unexpected.

We have revised this sentence to indicate that while KRAS expression is an important part of predicting KRAS dependency, it is not unexpected.

(11) I find Fig 2D hard to read, it is difficult to tell the difference between the circles and squares when the error bars are small.

We have changed the format in Fig 2D to improve readability.

(12) “many KRAS wild-type GI tract cancer patients also had low K20 scores, which we suspect may be due to false negatives in KRAS mutation detection” I think the authors are referring to TCGA patients here but based on Fig3B, the K20 scores are lower for KRAS wt cell lines from GI cancers as well. Could this be explained by tissue specific expression differences or copy number changes?

We thank the reviewer for this insightful question. It is possible that wild-type GI tract TCGA and CCLE samples had low K20 scores due to false negatives or tissue specific expression differences and copy number changes in KRAS.

(13) I’m curious why the copy number analysis was left out of the CORE TCGA analysis.

We used the subtypes provided in Supplementary Table 1 of Dinh et. al 2012, which does not contain the copy number variation information. We have now added analysis using Consensus Molecular Subtypes from Guinney et al (Nat Med, 2015).

(14) Authors should use the more widely accepted Consensus Molecular Subtypes (see Guinney, Nat Med, 2015) to analyze CORE samples. Moreover, line 381-387 are not results, and speculation on the part of the authors. Move to discussion.

We apologize for missing the more recent and widely adopted consensus subtypes for CORE and thank the reviewer for this suggestion. We added analyses using the Consensus Molecular Subtypes (Guinney et al, Nat Med, 2015) to Supplementary Figure 5F. Additionally, we moved text related to the CIMP-L and CIMP-H subtypes previously in lines 381-387 to the discussion.
