# Supplementary material for: An integrated model for predicting KRAS dependency
Source: PLoS Comput Biol. 2023 May 4;19(5):e1011095. doi: 10.1371/journal.pcbi.1011095 (PMC10187917; doi:10.1371/journal.pcbi.1011095)
Supplement: Attachment — Submitted filename: Response to Reviewers_230317.docx [file pcbi.1011095.s010.docx]

Dear Colleagues:

We thank the reviewers for their positive and constructive comments. We have addressed the concerns raised by the reviewers where experimentally possible. Changes from the original version are highlighted in the revised version of the manuscript and in the figures. Point-by-point summary follows:

Reviewer #1: This is a revised version of a manuscript by Pecot and others that focuses on developing a predictive signature for KRas modulation dependency across carcinomas. The motivation that biomarkers in addition to genetic status only for predicting KRas inhibition response remains well received. The authors have added to the manuscript to clarify previously unclear aspects.

We appreciate the positive comments of the reviewer as well as their constructive suggestions. Their prior comments, as well as the newer ones, have significantly improved the clarity of the work and the conclusions of our analyses.

1. The authors select six cell lines, two from each of the sensitive, intermediate, and refractory categories, with one being KRas WT (intermediate), and perform KRas siRNA dose responses and assay cell metabolic output (CellTiterGlo). On a positive note, the model seems to have correctly predicted the sensitivity of the KRas WT line.

a. I did not see any confirmatory experiments that KRas was indeed knocked down, or description of controls with non-targeting siRNA.

We thank the reviewer for this comment. We have previously characterized these siRNAs, which have consistently shown potent KRAS knockdown in all cell lines tested and are included in a prior publication (Pecot et al, Mol Cancer Ther, 2014). To further confirm knockdown of KRAS, we conducted quantitative real-time PCR on HCT116 cells to measure relative KRAS mRNA levels at 24 and 48hrs after treatment with both KRAS siRNAs used in the IC50 experiments. This data demonstrates 80-90% KRAS silencing and has been added to Panel A in Fig S3. We also added the negative control siRNA sequence to the methods. Experiments with the non-targeting siRNA did not show effects on knockdown or cell viability. We have clarified this in the text.

b. It is reported that the cells were allowed to grow in 96 well plates for six days following seeding / reverse transfection. This is quite long compared to typical dose response experiments, at least for small molecules (2-3 days), so some justification is warranted here.

We thank the reviewer for this observation. Because the mechanism of RNAi requires mRNA cleavage prior to protein degradation, we have observed the effects of RNAi targeting on cell viability is often delayed by ~48 hrs later than that observed with the mechanism of small molecule inhibitors. From our qPCR experiments, we found that 48 hours generally showed the most potent knockdown effects on KRAS mRNA, which eventually led to potent protein knockdown at around 72 hours. Thus, we found downstream cytotoxicity was most pronounced at 5-6 days after siRNA transfection, and indeed this is a few days later than often observed with small molecule inhibitors. In preliminary experiments, we conducted cytotoxicity readouts at 72, 96, 120, and 144 hours. We found that 144 hours showed the most robust IC50 curves across all cell lines, which is why we chose to take cytotoxicity readouts after 6 days. This is consistent with other RNAi experiments in the literature. We have clarified some of this justification in our methods section.

c. The authors have added Fig. S3A to better show a potential trend between K20 prediction score and response to KRas siRNA (GI50). Although a linear trend line is shown with r=0.62, the trend seems completely dependent on one cell line with a highly negative K20 prediction score (see also d below). Besides this data point, the trend is that there is essentially no dependence between the K20 prediction score and the siRNA GI50.

We thank the reviewer for pointing this out. Also, as described below in response to point d, we previously used incorrect K20 scores. To provide a more robust dataset, we ran 3 additional cell lines to obtain their GI50s, and we have updated Panel B in Fig S3 (previously Panel A) to include them in the correlation plot for GI50 and K20 prediction scores. The updated plot retains a statistically significant trend line of 0.57, and with the added cell lines provides further support that the K20 prediction score correlates well with KRAS dependence (GI50s).

d. Although which cell line referred to in point c above does not manifest from the plot, it may be SKCO1, from the sensitive cluster, based on the highly negative K20 Prediction score. This looks to be ~-1.6 from the plot, but is listed as -1.95 on Fig. 2D legend. Along these lines, the values for K20 scores in the Fig 2D legend do not seem to match those in S3A—reconciliation is needed.

We thank the reviewer for this astute observation, and we apologize for this mistake. Incorrect K20 scores had mistakenly been used when making the prior plot, and we are grateful for the opportunity to correct this. We have revised the graph in Panel B in Fig S3 to show the corrected K20 prediction scores. We have also individually labeled the cell lines to add clarity.

2. The authors apply the K20 panel to an independent dataset on lung cancer cell lines with KRas mutation treated with a mutant KRas inhibitor. Again, the preponderance of data seems to indicate most cell lines are resistant to the drug, yet have a wide range of K20 prediction scores between -1 and 0, which seems to indicate sensitivity to KRas inhibition based on the authors cutoff of ~1.4. How robust is the result to the lone data point on the far left?

We thank the reviewer for this comment. All the cell lines in Fig 2E were predicted to be non-refractory based on them being below the cutoff of 1.4. The point to the far left (with the smallest prediction score) was a cell line predicted to be sensitive with higher confidence, and indeed that cell line showed the lowest GI50 score. This dataset is also helpful in showing the dynamic range of KRAS-dependency across KRAS mutated cancers, which is now observed clinically with tumors demonstrating varying degrees of response to KRAS G12C inhibitors. For example, tumors with very low K20 predictions (e.g., less than -1) would be expected to have more significant responses to a KRAS inhibitor, however, tumors with K20 scores that approximate 1.4 may demonstrate only modest responses (or even just disease stability); and tumors with K20 scores >1.4 would be expected to demonstrate primary resistance. The ultimate utility of the K20 model will have to be evaluated using clinical tumors from patients that received KRAS inhibitors.

Other comments:

1. Fig S3 has two panel Bs.

We thank the reviewer for this observation. We have corrected these panels.

2. It is still logically unclear to me why to make three clusters of cell lines when a binary outcome is modeled.

We thank the reviewer for this comment. First, we began our model development by following the methods previously described in McDonald et al (Cell, 2017) to apply k-mean clustering (k=3), which we also think accurately represents the clinical response categories.

However, in the clinic, response to drugs is usually classified as “response (complete or partial)”, “stable disease” and “disease progression”. In the clinical setting, the primary outcome measurement is response rate, but many trials use clinical benefit as well, which includes both responders and those patients with stable disease. An example of this would be the recent colon cancer data demonstrating KRAS G12C inhibitors largely only result in disease control (stable disease), while achieving minimal response rates (Fakih et al, The Lancet, 2022). In this manuscript, we included the intermediate k-mean clustering group (which may reflect patients that will experience stable disease) as “non-refractory” to also reflect potential benefit from KRAS inhibitors. Thus, tumors with a K20 score of <1.4 would be anticipated to result in either a response or stable disease, and those with scores >1.4 would likely demonstrate primary resistance and disease progression.

3. Page 7, line 131, it is confusing to introduce the K20 score and 19 genes before development of the model (I am not sure if that’s what the authors intended but it confused me).

We thank the reviewer for this comment and appreciate the suggestion. We have revised the sentence and removed the model details from the beginning of the results section to remove this confusion and add clarity.

4. The authors address prior modeling work on KRas sensitivity gene signatures (a point raised by Reviewer 4) by saying a pan-cancer signature is needed, which has not been done. However it is not clear why cancer-type-specific models would be suboptimal. In fact, they may be better at predicting due to tissue type differences across cancer types. In their response to Reviewer 2, the authors say there simply was not enough data yet to build robust classifiers in a cancer specific way, which seems contradictory here.

We thank the reviewer for this comment and agree it would eventually be optimal if cancer-type-specific models could be made available. However, the approach we took used cell lines with both transcriptome and KRAS dependency data available. Some cancer types had data available for only one cancer cell line, making it impossible to build a prediction model. Even lung cancer, which had the largest amount of available data (36 cell lines), was not sufficiently powered to build a robust classifier without overfitting the data. Therefore, we think combining data from different cancer types was necessary to increase the statistical power.

5. The authors select genes that had variations (SD > 0.5) in lung, pancreatic and colorectal cancer mRNA datasets in TCGA. Variations with respect to what? This was not clear from the methods.

We thank the reviewer for this comment. The variation of gene expression is referring to statistical variance here, which measures variability from the average expression. We believe genes with low expression variance in tumors should not be included in the model because it’s harder to distinguish noise from signal when the variance is small. We have added clarifications on this to the methods section under “TCGA and CCLE Joint statistic calculation.”

6. It was not described in the methods how the K20 score was actually calculated from the 20 features. Is it a dot product of the feature value vector and their coefficients?

We appreciate this observation and have clarified how this is calculated in the methods section. We have also included the coefficient in Supplementary Table 1.

7. The pages and line numbers referenced in the rebuttal letter do not line up with the revised paper.

We thank the reviewer for this comment. We will request direction from the editor with how to address this discrepancy.

Reviewer #3: Regarding the 5th comment, gene expression index was just normalized by sequencing depth, it should be RPM. RPKM is the normalized expression index with sequencing depth and gene size.

We apologize for this confusion. It is also normalized to gene size (and is therefore correctly written as RPKM). We have revised this in the manuscript to make it clear.
